# Heterozygosity levels and estimation of self‐fertilization in an invasive species

**DOI:** 10.1002/ece3.6885

**Published:** 2020-11-20

**Authors:** Philip W. Hedrick

**Affiliations:** ^1^ School of Life Sciences Arizona State University Tempe AZ USA

The article by Winkler et al. (2019) about invasive Sahara mustard (*Brassica tournefortii*) could prove useful in understanding the spread of invasive, non‐native species. For invasive species, self‐fertilization is thought to be an advantageous characteristic because a single individual can start a new population without need of other individuals for reproduction. As a result, self‐fertilizing, invasive species are thought to be particularly hard to control because they are so effective in spreading and colonizing new areas. Winkler et al. (2019) stated that Sahara mustard was facultatively autogamous, that is, it primarily self‐fertilizes but that outcrossing is possible. They then estimated that the self‐fertilization rate *S* in a number of Sahara mustard populations was very high, that is, *S* was about 0.9 (the rate of outcrossing was only around 0.1), making the potential for population increase quite elevated.

However, the observed heterozygosity *H*_O_ values and expected heterozygosity *H*_E_ values, based on Hardy–Weinberg proportions within subpopulations, given in Winkler et al. (2019) do not correspond with high self‐fertilization rates. Table 1 gives the observed and expected heterozygosity values for the three genetic clusters of populations found by Winkler et al. (2019) and given in their Table 1 (and table 3.2 in Winkler, 2017), as well as their estimates of inbreeding *F*_IS_ and *S*.

**TABLE 1:** The observed heterozygosity *H*_O_ and expected heterozygosity *H*_E_ for the three clusters of populations (*N* indicates the number of populations in the clusters) identified by Winkler et al. (2019)

Cluster	*H*_O_	*H*_E_	Winkler et al. (2019)		PSA	
			*F*_IS_	*S*	*F*_IS_	*S*
1 (*N* = 47)	0.058	0.070	0.84	0.91	0.17	0.29
2 (*N* = 4)	0.056	0.056	0.78	0.88	0.00	0.00
3 (*N* = 1)	0.055	0.036	0.79	0.88	−0.55	−2.44

Note

Also given for these three clusters is the level of inbreeding *F*_IS_ and self‐fertilization *S* estimated by Winkler et al. (2019) and the level of inbreeding and self‐fertilization estimated by using these heterozygosity values and the traditional Population Structure Approach (PSA)

In an initial response to this letter, Winkler et al., suggested that they used *H_S_*, the expected proportion of heterozygotes within subpopulations, instead of *H_E_*, to calculate *F*_IS_. Of course, this is what *H_E_* within subpopulations should be and what Winkler (2017) stated it was. If *H_E_* and *H_S_* are different, it is not clear how the values of *H_E_* in Winkler et al. (2019) and Winkler (2017) were calculated. In their initial response, they also cited two articles in which *H_S_* is used to indicate the “standardized heterozygosity”. This is a different measure and should not be used to estimate *F*_IS_, suggesting that there was potential confusion between these measures, both termed *H_S_*, on their part.

Winkler et al. (2019) estimated that the levels of inbreeding in the three clusters range only from 0.78 to 0.84 (with the consequent level of self‐fertilization ranging only from 0.88 to 0.91), making the levels of these measures very similar in the three different clusters. With such high self‐fertilization, it would be expected that *H*_O_ would be only about 20% of *H*_E_. However, in Table 1 for Cluster 1, *H*_O_ is 83% of *H*_E_, for Cluster 2, *H*_O_ = *H*_E_, and for Cluster 3, *H*_O_ is 153% of *H*_E_. The value of *S* = 0.88 given for Cluster 3 (with only one population) but with *H*_O_ = 0.055 and *H*_E_ = 0.036 is particularly unexpected.

The traditional approach of estimating *F*_IS_ from genotypic data is to use the equation *F*_IS_ = 1–*H*_O_/*H*_E_ (Weir & Cockerham, 1984), which Jarne and David (2008) term the Population Structure Approach (PSA). With this estimate of *F*_IS_, the proportion of self‐fertilization in a mixed‐mating system can then be estimated as *S* = 2*F*_IS_/(1 + *F*_IS_) (Jarne & David, 2008). Using these equations and the heterozygosity data from Winkler et al. (2019), *F*_IS_ and *S* are given on the right side of Table 1. Obviously, these estimates of *F*_IS_ and *S* are completely incongruous with those given in Winkler et al. (2019), that is, for Cluster 1, there appears to be some small amount of self‐fertilization (*S* = 0.29), for Cluster 2, there appears to be random mating (*S* = 0.00), and for Cluster 3, with only one population the value suggests very high mating with some genetically different group (*S* = −2.44).

Further genetic detail about the populations used in this study was given in table S3.1 of Winkler (2017). From that table, the 52 populations can be divided into two groups, 23 with *H*_O_ > *H*_E_ and 29 with *H*_O_ < *H*_E_ (there were no populations with similar observed and expected heterozygosities). Table 2 gives the averages within these two groups and the level of observed heterozygosity *H*_O_ is virtually the same in the two groups, 0.058 in the first and 0.057 in the other. On the other hand, the average level of expected heterozygosity *H*_E_ is very different in the two groups, 0.038 in the first and 0.094 in the second, even though populations of both Cluster 1 and 2 are in each category.

**TABLE 2:** The level of observed heterozygosity *H*_O_ and expected heterozygosity *H*_E_ in the 52 populations in Winkler (2017) separated into two groups, that is, those with either with *H*_O_ > *H*_E_ or *H*_O_ < *H*_E_, where *N* is the number of populations in the cluster with the different heterozygosity characteristics

Cluster	*H*_O_ > *H*_E_			*H*_O_ < *H*_E_		
	*N*	*H*_O_	*H*_E_	*N*	*H*_O_	*H*_E_
1	20	0.058	0.038	27	0.057	0.095
2	2	0.055	0.044	2	0.057	0.076
3	1	0.055	0.036	0	—	—
Average		0.058	0.038		0.057	0.094

It is not clear why there is a large difference between *H_E_* in these groups that have very similar *H_O_* values but it suggests some problem in the calculation of *H_E_*. If the traditional PSA approach is used to estimate *F*_IS_ and *S*, then in the group with *H*_O_ > *H*_E_, *F*_IS_ = −0.53 and *S* = −2.22 and in the group with *H*_O_ < *H*_E_, *F*_IS_ = 0.39 and *S* = 0.56. These values are clearly unlike any estimates given in Winkler (2017) or Winkler et al. (2019). Given these puzzling observations, and because a biological basis for these patterns is not obvious, the basic data used and their analysis need to be re‐examined.

The differences in Table 1 in the level of the estimates of inbreeding and self‐fertilization from Winkler et al. (2019) and the traditional PSA (Jarne & David, 2008) are quite baffling. The further comparisons showing the much higher levels of expected heterozygosities in 29 populations with *H*_O_ < *H*_E_ compared to the 23 populations with *H*_O_ > *H*_E_ in Table 2, and the estimates of inbreeding and selfing from them, suggests some calculation or analysis error. Because of the importance of the level of self‐fertilization in invasive, non‐native species, it is critical that the bases of these perplexing disparities be clarified.

## CONFLICT OF INTEREST

None declared.

## AUTHOR CONTRIBUTION

**Philip W. Hedrick:** Conceptualization (lead); Writing‐original draft (lead).
